# Quantitative *T*_2_^*^ assessment of acute and chronic myocardial ischemia/reperfusion injury in mice

**DOI:** 10.1007/s10334-012-0304-0

**Published:** 2012-02-11

**Authors:** Eissa N. E. Aguor, Fatih Arslan, Cees W. A. van de Kolk, Marcel G. J. Nederhoff, Pieter A. Doevendans, Cees J. A. van Echteld, Gerard Pasterkamp, Gustav J. Strijkers

**Affiliations:** 1 Department of Cardiology, University Medical Center Utrecht (UMCU), Utrecht, The Netherlands; 2 Laboratory of Experimental Cardiology, University Medical Center Utrecht (UMCU), Heidelberglaan 100, 3584 CX Utrecht, The Netherlands; 3 The Netherlands Heart Institute, Utrecht, The Netherlands; 4 Biomedical NMR, Department of Biomedical Engineering, Eindhoven University of Technology, Eindhoven, The Netherlands

**Keywords:** Cardiovascular magnetic resonance, Myocardial infarction, *T*_2_^*^, LGE, Mouse, Fibrosis, Hemorrhage

## Abstract

**Object:**

Imaging of myocardial infarct composition is essential to assess efficacy of emerging therapeutics. *T*_2_^*^ mapping has the potential to image myocardial hemorrhage and fibrosis by virtue of its short *T*_2_^*^. We aimed to quantify *T*_2_^*^ in acute and chronic myocardial ischemia/reperfusion (I/R) injury in mice.

**Materials and methods:**

I/R-injury was induced in C57BL/6 mice (*n* = 9). Sham-operated mice (*n* = 8) served as controls. MRI was performed at baseline, and 1, 7 and 28 days after surgery. MRI at 9.4 T consisted of Cine, *T*_2_^*^ mapping and late-gadolinium-enhancement (LGE). Mice (*n* = 6) were histologically assessed for hemorrhage and collagen in the fibrotic scar.

**Results:**

Baseline *T*_2_^*^ values were 17.1 ± 2.0 ms. At day 1, LGE displayed a homogeneous infarct enhancement. *T*_2_^*^ in infarct (12.0 ± 1.1 ms) and remote myocardium (13.9 ± 0.8 ms) was lower than at baseline. On days 7 and 28, LGE was heterogeneous. *T*_2_^*^ in the infarct decreased to 7.9 ± 0.7 and 6.4 ± 0.7 ms, whereas *T*_2_^*^ values in the remote myocardium were 14.2 ± 1.1 and 15.6 ± 1.0 ms. Histology revealed deposition of iron and collagen in parallel with decreased *T*_2_^*^.

**Conclusion:**

*T*_2_^*^ values are dynamic during infarct development and decrease significantly during scar maturation. In the acute phase, *T*_2_^*^ values in infarcted myocardium differ significantly from those in the chronic phase. *T*_2_^*^ mapping was able to confirm the presence of a chronic infarction in cases where LGE was inconclusive. Hence, *T*_2_^*^ may be used to discriminate between acute and chronic infarctions.

## Introduction

Despite recent advances in therapeutics and diagnostics, morbidity and mortality after myocardial infarction (MI) remains a great socio-economic burden in Western societies. Early identification of patients with sub-optimal cardiac recovery is of great importance to improve clinical management. For this reason, both functional and structural characterization of the infarcted myocardium is important for risk stratification of patients after MI.

Technological advances in cardiovascular magnetic resonance (CMR) have demonstrated different ways to track and characterize MI at different time points [1–6]. CMR has been applied to analyze inflammation, edema, necrosis, fibrosis, left-ventricular (LV) remodeling and metabolism in MI [7–15]. Pathological changes in the injured myocardium, such as the presence of fibrosis, hemorrhage and edema, influence the signal intensity on *T*_1_-and *T*_2_-weighted imaging [7, 16–18]. CMR is thus considered as one of the most important imaging tools in the assessment of the heart after MI.

Typically, *T*_1_-weighted imaging is performed some time after injecting a gadolinium-based contrast agent to delineate injured myocardium in a late gadolinium enhancement (LGE) scan [19]. The enhancement mechanism of infarcted tissue is related to the extent of the extracellular volume, which is associated with the delayed washout relative to the healthy myocardium. LGE is widely used and, without competition, considered the gold standard to assess infarct size. Nevertheless, there are certain limitations of LGE related to pathological processes after the onset of infarction. In the acute and sub-acute phase after successful reperfusion of the ischemic myocardium, the transmural infarct extent is not stable and an overestimation of infarct size has been reported [20]. In the chronic phase, excessive and heterogeneous collagen deposition during scar maturation may hamper signal enhancement by LGE.

In view of the above limitations, several alternative contrast mechanisms are explored to study the pathophysiological changes in the injured myocardium. The sensitivity of the *T*_2_ relaxation time to myocardial water content has led to the application of *T*_2_-weighted CMR protocols to image myocardial edema as an indicator of inflammation and necrosis in acute MI [13, 15, 21–24]. *T*_2_^*^ contrast has been explored as a tool to diagnose the presence of hemorrhage and myocardial iron [1–5, 25–28]. Recently, it was demonstrated that *T*_2_^*^ has the potential to image fibrosis in the infarct zone using an ultra-short echo time (UTE) technique [29].

In this paper, we hypothesize that the structural and pathological changes in acute and chronic MI, such as the presence and development of myocardial hemorrhage and fibrotic tissue progressively influence *T*_2_^*^ values in the infarcted myocardium. To investigate this, we quantified *T*_2_^*^ using a *T*_2_^*^-mapping protocol longitudinally in acute and chronic ischemia/reperfusion (I/R) infarcts in mice. We explored the utility of *T*_2_^*^ mapping as a complementary tool to LGE to characterize the infarcted myocardium.

## Materials and methods

### Experimental design

C57Bl6/J mice (*n* = 23, body weight = 20–25 g) were used in this study. CMR measurements were performed at baseline (BL), 1 day (acute infarct), 7 and 28 days (chronic infarct) after induction of MI by 30 min transient ligation of the left coronary artery (LCA) (*n* = 9). A sham-operated group (*n* = 8) served as control. The CMR protocol for the infarct group consisted of a multi-gradient-echo quantitative *T*_2_^*^-mapping sequence, traditional LGE to assess the location and size of the infarct, and gradient-echo Cine imaging to determine cardiac function. The sham-operated (control) group underwent gradient-echo Cine imaging only to evaluate cardiac function. In the first group of nine mice with MI, one mouse died at day 28 during the CMR scan. Another three mice of this group presented a small infarct on LGE images at day 1, but no chronic myocardial infarct at day 7 and 28 (as exemplified in Fig. 3). A separate set of mice with MI (*n* = 6) were killed either at day 1 (*n* = 2), 7 (*n* = 2), or 28 (*n* = 2) for the histopathological characterization of myocardial tissues. All animal experiments were performed in accordance with the national guidelines on animal care and with prior approval by the Animal Experimentation Committee of Utrecht University.

### Mouse model of myocardial I/R injury

Mice underwent myocardial I/R surgery as previously described [30]. Briefly, mice were anesthetized with a mixture of Fentanyl (Jansen-Cilag) 0.05 mg/kg, Dormicum (Roche) 5 mg/kg, and medetomidine 0.5 mg/kg through an intraperitoneal injection. A core body temperature of about 37°C was maintained during surgery by continuous monitoring with a rectal thermometer and an automatic heating blanket. Mice were intubated and ventilated (Harvard Apparatus Inc, Holliston, MA) with 100% oxygen. The LCA was ligated for 30 min with an 8-0 Ethilon (Ethicon) suture with a section of polyethylene-10 tubing placed over the LCA. Ischemia was confirmed by bleaching of the myocardium and ventricular tachyarrhythmia. Reperfusion was initiated by release of the ligature and removal of the polyethylene-10 tubing. In sham-operated animals, the suture was placed beneath the LCA without ligation. Reperfusion of the endangered myocardium was characterized by typical hyperemia in the first few minutes. The chest wall was closed, and the animals subcutaneously received Antisedan (Pfizer) 2.5 mg/kg, Anexate (Roche) 0.5 mg/kg, and Temgesic (Schering-Plough) 0.1 mg/kg.

### CMR protocol

A vertical 9.4 T and 89-mm-diameter bore scanner was used, equipped with 1,500 mT/m gradients (Bruker BioSpin GmbH, Ettlingen, Germany). Mice were anesthetized with 1.5–3 vol% isoflurane in a 2:1 mixture of air (0.3 l/min) and oxygen (0.15 l/min). The mice were put on a custom-built cradle and positioned inside a 3-cm-diameter quadrature volume coil. During the CMR examination anesthesia was maintained at 1.5–2.5 vol% isoflurane to keep the respiratory rate at 50–60 bpm. We did not use ECG leads for cardiac triggering but derived both cardiac and respiratory motion signals from a pressure pad placed under the chest of the mouse.

**Fig. 1:**
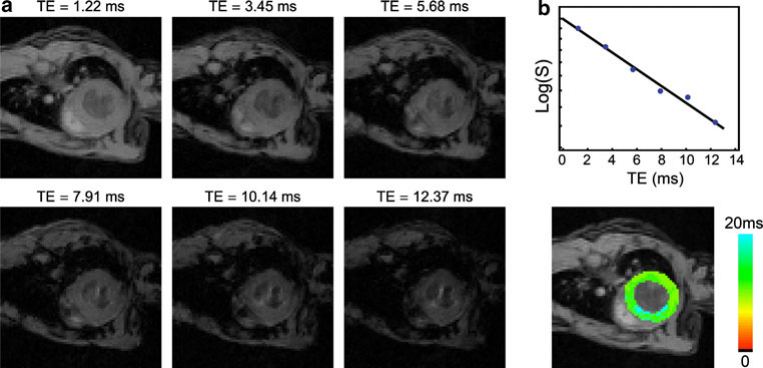
Representative series of cardiac images with varying TE and corresponding *T*_2_^*^ map. **a** Gradient-echo CMR with varying TE for a mid-ventricle short-axis slice of a representative mouse at baseline. **b** *Top* signal intensity *S* in the myocardium as function of TE. The *solid line* is an exponential fit to the data. *Bottom* *T*_2_^*^ map. *T*_2_^*^ in the LV myocardium at end-diastole is *color-coded* between 0 and 20 ms according to the pseudo *color scale* on the *right*. The background image is a regular gradient echo image with TE = 1.22 ms

The CMR protocol consisted of the following steps. After global shimming and RF pulse calibration, 2- and 4-chamber view scout scans were made to plan a single mid-cavity short-axis slice. Cine imaging was performed with a retrospectively-triggered (self-gated) gradient-echo sequence [31], with the following parameters: TR = 6.8 ms, TE = 1.9 ms, number of movie frames = 15, slice thickness = 1 mm, matrix = 256 × 256, field-of-view = 3 × 3 cm^2^. Because of the slight difference of hearts size between mice and the dilation of each heart after infarction, seven to nine short-axis slices with inter-slice distance of 1 mm and no slice gap were measured to cover the heart from apex to base. Based on the Cine short-axis images, a mid axial slice was selected that included an area of remote viable tissue as well as a substantial infarct area. *T*_2_^*^ mapping was performed in this slice using a cardiac-triggered multi-gradient-echo sequence with the following parameters: TR = *R*–*R* interval, TE = 1.22, 3.45, 5.68, 7.91, 10.14, and 12.37 ms, slice thickness = 1 mm, matrix = 128 × 128, field-of-view = 3 × 3 cm^2^. The trigger delay was chosen in the mid-diastolic rest period of the heart cycle observed in the Cine series. The acquisition window of less than 13 ms resulted in images, which were sufficiently co-registered for the different echo times to allow for a pixel-wise *T*_2_^*^ determination (Fig. 1) [32]. LGE scans were performed in the same slice with a cardiac-triggered inversion-recovery segmented gradient-echo sequence, with the following parameters: TR = 5.8 ms, TE = 2.2 ms, number of segments = 16, slice thickness = 1 mm, matrix = 256 × 256, field-of-view = 3 × 3 cm^2^. The inversion time (TI) was optimized to null the signal from remote myocardium [33, 34]. Gadobutrol (Gadovist, Bayer Schering Pharma AG, Berlin, Germany) dose of 1 mmol/ml was used. To reduce volume related variability, contrast agent was diluted 1:4 with a standard saline solution. This dilution resulted in administered weight-adapted volume of 2 μl/g body weight, resulting in an equivalent dose of 0.5 mmol/kg body weight [33]. The contrast agent was injected intravenously through the tail vein, using a 24-gauge catheter.

### CMR data analysis

*T*_2_^*^ maps were generated in Mathematica 7 (Wolfram Research, Champaign, IL, USA) by pixel-wise fittings of the multi-gradient-echo signal intensities to the equation $S = S_{0} {\text{e}}^{{ - {\text{TE}}/T_{2}^{*} }}$. The LV myocardium was manually segmented by drawing epicardial and endocardial contours, excluding the papillary muscles. LGE-based single-slice infarct size is reported as percentage of the LV myocardial cross-sectional area. A region of interest in the infarct in the myocardial free wall (infarct ROI) was drawn based on gadolinium enhancement and presence of akinetic myocardium. A second ROI was drawn in the non-enhancing remote tissue in the septum (remote ROI). For the control mice, ROIs were drawn in the septum and the free wall. These ROIs were drawn within the zone free from susceptibility artifacts induced by lungs. *T*_2_^*^ values are reported as group average and standard deviation of the mean *T*_2_^*^ in the ROIs. Cine images were analyzed with Qmass digital imaging software (Medis, Leiden, The Netherlands). Semi-automatic segmentation was used to determine end-diastolic volume (EDV), end-systolic volume (ESV), wall thickness (WT) and systolic wall thickening (SWT). Ejection fraction (EF) was calculated as 100%(EDV − ESV)/EDV.

### Histopathology

For histological evaluation a 1.5-mm-thick slice containing infarcted myocardium was fixed in 4% formalin and paraffin-embedded. Hereafter, 5-μm-thick sections were mounted on glass slides and stained with Picrosirius red staining for collagen, Prussian blue staining for iron, and Hematoxylin-Eosin (HE) for cellular structures. The stained sections were photographed with a digital image microscopy using white or polarized light.

### Statistical analysis

All statistical analysis was evaluated using (SPSS 17 for Windows; SPSS). Results were expressed as mean ± standard deviation for each group of animals. An independent two-tailed sample *t* test and the Mann–Whitney exact two-tailed *U* test were performed to compare mean values in subjects for each time point post MI versus baseline. Results were considered statistically significant for a *p* value of less than 0.05. The correlation analysis of *T*_2_^*^ values against WT was performed using linear regression.

## Results

An image series of a mid-axial slice with varying TE for a representative mouse at baseline is shown in Fig. 1a. The LV myocardium was well co-registered for the different images. Signal loss due to susceptibility differences was occasionally observed for the images with the longest TE. Pixel-wise fitting of the signal intensities in the LV myocardium as function of TE resulted in a *T*_2_^*^ map as shown in Fig. 1b. *T*_2_^*^ values were homogeneous throughout the LV myocardial wall and consistent between mice, with a mean *T*_2_^*^ value of 17.1 ± 2.0 ms in the free wall.

**Fig. 2:**
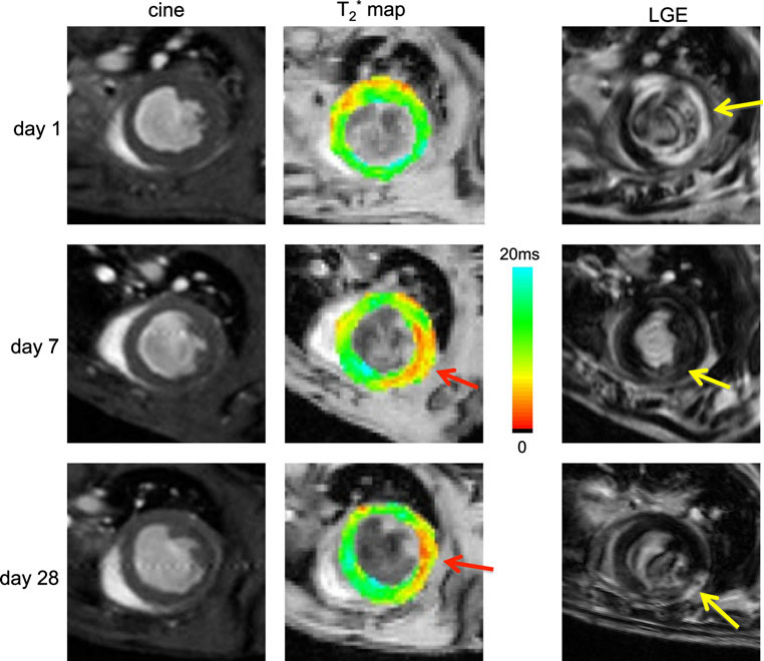
Mid-ventricle short-axis slice of a mouse heart at 1, 7, and 28 days after I/R myocardial infarction. *Left* Cine images at end-diastole. At day 1 wall thickness in the free wall is increased, whereas at days 7 and 28 the free wall decreased in thickness. *Middle* corresponding *T*_2_^*^ maps color-coded from 0 to 20 ms. *T*_2_^*^ is decreased in the infarct in the free wall at day 7 and 28 (*red arrows*). *Right* corresponding LGE images. *Yellow arrows* point to the location of the infarct. Infarct location was confirmed by the presence of an akinetic area and locally reduced wall thickening in Cine imaging

Figure 2 displays a collection of images, consisting of LGE, *T*_2_^*^ maps, and Cine images at diastole, for a representative mouse at day 1, 7 and 28 after induction of the MI. On day 1, LGE resulted in a homogeneous enhancement of the infarct in the mid-anterior and mid-anterolateral wall, whereas on days 7 and 28 enhanced area was smaller, heterogeneous, and difficult to detect. Corresponding *T*_2_^*^ maps revealed a different picture. At day 1, a significant change in *T*_2_^*^ could be detected in the LGE-based infarct area. Further, at days 7 and 28, *T*_2_^*^ dramatically decreased, particularly in the mid-anterolateral region indicating the existence of a significant infarct. The presence of an infarct was further corroborated by the Cine images, which showed increased WT in the mid-anterior and mid-anterolateral wall at day 1, followed by progressive thinning below baseline levels at days 7 and 28, respectively.

**Fig. 3:**
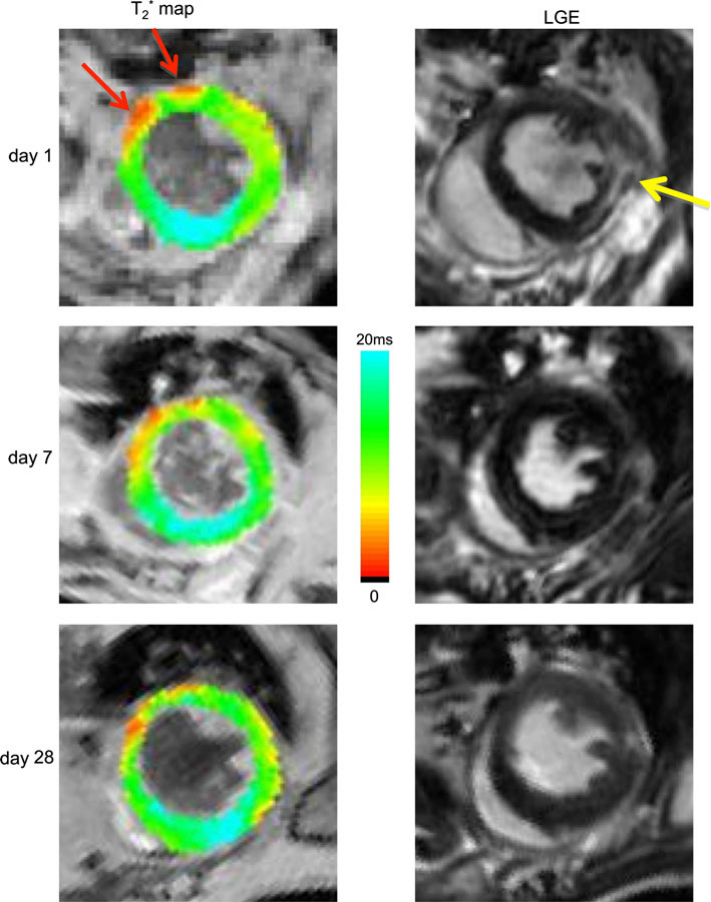
*T*_2_^*^ maps and LGE of a mouse with a small LGE positive area at day 1 but no detectable infarct at later days. LGE showed enhancement in the mid-anterior wall at day 1 (*yellow arrow*). Enhancement, however, was absent at 7 and 28 days. In parallel, *T*_2_^*^ values in the infarct area were maintained at baseline levels. *T*_2_^*^ maps displayed spurious lowering in areas with no infarct related to susceptibility artifacts (*red arrows*)

Figure 3 shows LGE and *T*_2_^*^ maps of one of three mice with a small infarct in the mid-anterolateral wall, as indicated by a small area of LGE at day 1. Gadolinium enhancement could not be detected anymore at days 7 and 28. In the first group of *n* = 9 mice with MI, *n* = 3 mice displayed this atypical behavior. Presumably, LGE at day 1 revealed a small area at risk that could not be detected in the chronic phase. Importantly, *T*_2_^*^ values in the mid-anterolateral wall also remained unaffected throughout infarct healing and did not deviate from baseline values.

**Fig. 4:**
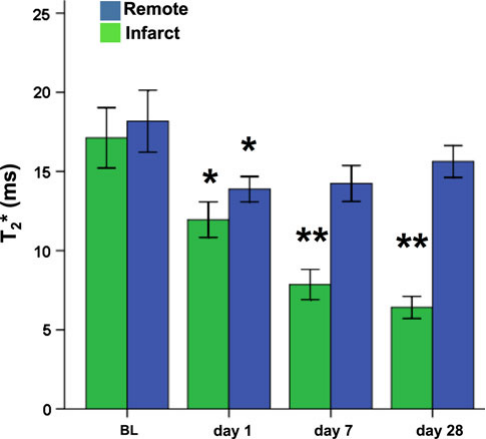
Quantitative *T*_2_^*^ values in the infarct and remote myocardium at baseline (BL) and up to 28 days after surgery. *n* = 5 mice, *significantly different from baseline (*p* < 0.05), and **significantly different from baseline (*p* < 0.005)

Quantitative *T*_2_^*^ values (*n* = 5 mice, Fig. 4) were dynamic throughout infarct development. In the infarct, quantitative *T*_2_^*^ values decreased significantly (*p* < 0.05) from baseline (17.1 ± 2.0 ms) to day 1 (12.0 ± 1.1 ms) followed by a further decrease (*p* < 0.005) both at day 7 (7.9 ± 1.0 ms) and day 28 (6.4 ± 0.7 ms). In addition, the mid-septal (remote) area showed a significant (*p* < 0.05) decrease of *T*_2_^*^ values from baseline (18.2 ± 2.0 ms) to day 1 (13.9 ± 0.8 ms), then slightly recovered at day 7 (14.2 ± 1.1 ms) and day 28 (15.6 ± 1.0 ms).

**Fig. 5:**
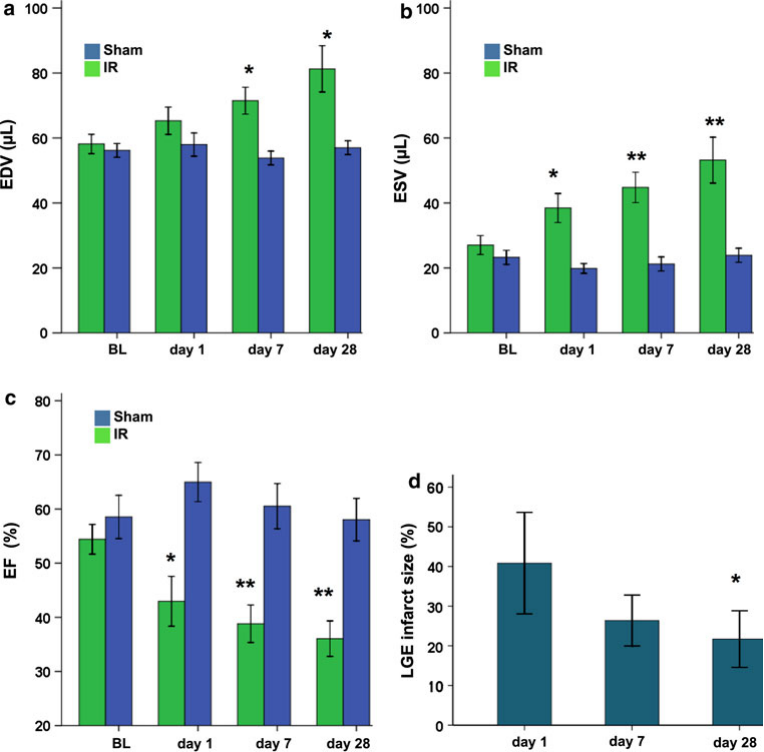
Left ventricle functional parameters and infarct size for infarct (*n* = 5) and sham-operated (*n* = 8) groups at baseline (BL) and up to 28 days after surgery. **a** End diastolic volume. **b** End systolic volume. **c** Ejection fraction. **d** Single-slice infarct size. *Significantly different from baseline (*p* < 0.05), and **significantly different from baseline (*p* < 0.005)

Global cardiac function of both infarct and sham-operated groups was assessed by Cine imaging (Fig. 5a–c). The infarct group displayed a progressive and significant increase in EDV and ESV, indicative for a considerable dilation of the heart due to the infarct. For the sham-operated group no significant changes in EDV and ESV upon time after surgery were observed. The infarct group presented a decline in EF from 54 ± 3% at baseline to 39 ± 2%, 28 days after surgery (Fig. 5c). For the sham-operated group, EF was slightly elevated at day 1; however, it normalized at day 7 and 28 after surgery. Group-averaged (*n* = 5) infarct sizes from LGE analysis of a mid-ventricular slice as function of days after surgery are presented in Fig. 5d. At day 1, LGE revealed an average infarct size of 41 ± 6%, dropping to 26 ± 3 and 22 ± 4 % (*p* < 0.05, compared to day 1) of the LV area at days 7 and 28, respectively, which could be related to infarct resorption over time [35].

**Fig. 6:**
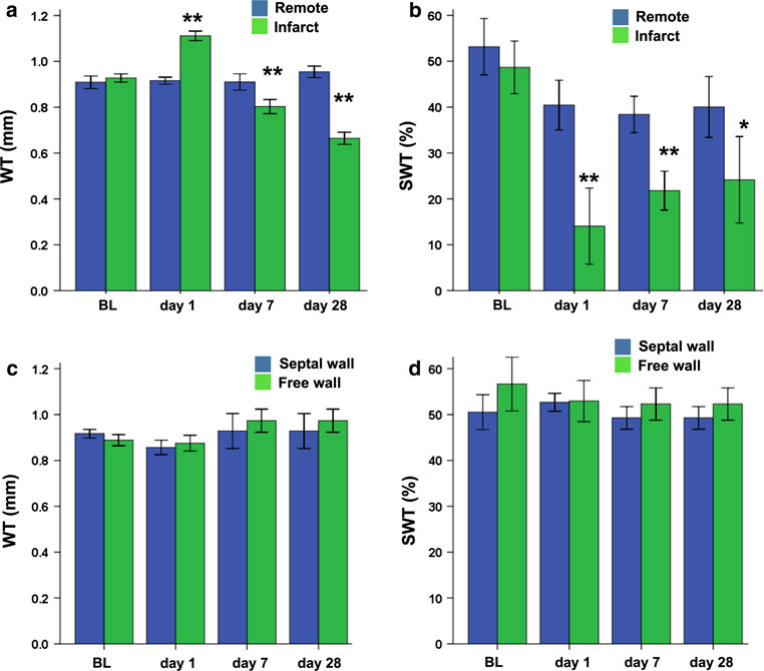
Regional wall thickness (WT) and systolic wall thickening (SWT) for infarct (*n* = 5) and sham-operated (*n* = 8) groups. **a** WT for infarct group in remote and infarct wall. **b** SWT for infarct group in remote and infarct wall. **c** WT for sham-operated group in septal and free wall. **d** SWT for sham-operated group in septal and free wall. *Significantly different from baseline (*p* < 0.05), and **significantly different from baseline (*p* < 0.005)

**Fig. 7:**
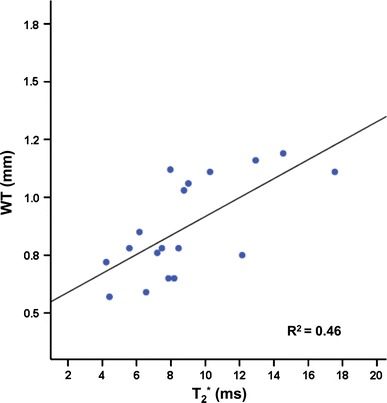
Correlation between infarct wall thickness (WT) and *T*_2_^*^

In the infarct group, WT in the free wall (infarct area) showed a significant (*p* < 0.005) transient increase day 1 after MI, followed by progressive thinning (Fig. 6a). Decreased WT in the infarct paralleled a decrease of the quantitative *T*_2_^*^ values (Fig. 4). The mid-septal (remote) area in the infarct group showed no significant changes in WT. In line with the increased WT of the free wall at day 1, the SWT, as a measure of local systolic function, also displayed a transient depression 1 day after surgery, only partially recovering at days 7 and 28 (Fig. 6b). Also, SWT of the remote area was slightly depressed at all time points compared to baseline. The sham-operated group revealed no significant changes in both WT and SWT as function of days after surgery (Fig. 6c, d). WT in the infarct correlated positively and significantly with *T*_2_^*^ (Fig. 7). Thus, the chronic MI is characterized by a lower *T*_2_^*^ value compared to the acute MI.

**Fig. 8:**
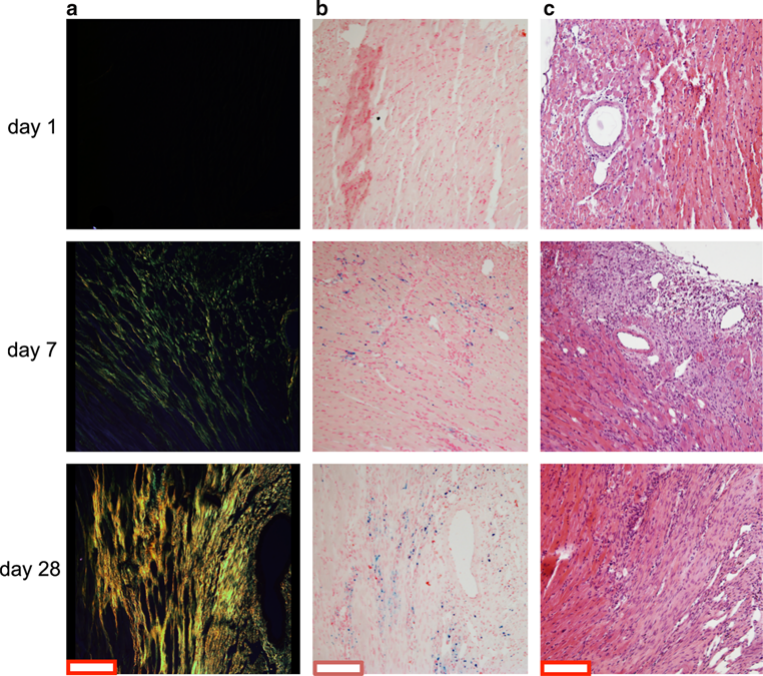
Representative histological images of infarct myocardial tissue at day 1, 7 and 28 post MI. **a** Picrosirius red. **b** Prussian-blue. **c** Hematoxylin-Eosin. *Scale bar* equals 200 μm

Since *T*_2_^*^ changes are related to the structural and compositional changes in the infarct myocardial tissue, we performed histology at each time point after I/R injury. Figure 8 shows images of three representative mice at day 1, 7 and 28 after MI. Picrosirius staining (Fig. 8a) revealed no collagen deposits in the infarct area at day 1. At day 7 substantial collagen deposition was observed, followed by a significant increase at day 28. Sections stained with Prussian Blue showed scattered spots of iron as early as 1 day after MI (Fig. 8b). Iron deposition became more prominent at day 7 and further increased at day 28. Finally, on sections stained with HE (Fig. 8c) the infarct area on day 1 was characterized by infiltration of inflammatory cells.

## Discussion

In this study we aimed to systematically study quantitative changes in *T*_2_^*^ of the mouse myocardium up to 28 days post I/R injury and to evaluate its added value in characterizing chronic infarcts in comparison to commonly applied LGE and Cine imaging. Combining different CMR techniques, if interpreted carefully, are a potential advantage in the evaluation of the pathophysiological changes in the injured myocardium. Baseline *T*_2_^*^ values were comparable to T_2_ values found at 9.4 T [36] and 11.75 T [37], which suggests that decay due to static magnetic field inhomogeneities is largely absent in the healthy myocardium. We found that quantitative *T*_2_^*^ values decreased progressively during infarct development. Throughout the observation period, the local decrease of *T*_2_^*^ in the infarct was accompanied by globally and locally reduced ventricular function (Figs. 4, 5, 6, 7). The reduction in *T*_2_^*^ with infarct age could be explained by the presence of iron (Fig. 8a) [3, 38]. Additionally, the formation of significant amounts of collagen (Fig. 8b) may contribute to the progressive decline of *T*_2_^*^ with infarct age [29].

LGE imaging is currently unchallenged the gold standard technique to assess infarct size. Nevertheless, in certain cases when LGE imaging is inconclusive, additional characterization of the infarct by *T*_2_^*^ imaging could provide complementary information. In the acute phase after I/R injury LGE-based infarct size may be inaccurate [39], when there is loss of cell membrane integrity [16], an inflammatory response [40, 41], necrosis, hemorrhage, micro-vascular obstruction (MVO) and edema. Indeed, for the acute infarcts at day 1 we found a large LGE-based infarct size accompanied by increased wall-thickness, suggesting the presence of edema. We did not observe a transient increase in *T*_2_^*^ at day 1 by the presence of this edema, as previously observed with *T*_2_-weighted imaging [24]. The effect of edema on *T*_2_^*^ is apparently lower than the decrease of *T*_2_^*^ induced by iron or collagen (Fig. 8). This observation is critical in the clinical setting where early assessment of viability is significantly hampered by the presence of infarct-induced edema. *T*_2_^*^-imaging may be a tool to circumvent this inevitable problem after acute MI.

In the chronic infarct, the formation of scar tissue could be responsible for a reduced distribution volume for the extracellular contrast agent. In turn, reduced distribution volume leads to an apparent reduction in infarct size and inhomogeneous enhancement (Figs. 2, 5). Additionally, the extremely low *T*_2_^*^ in the infarct (Fig. 2), which locally decreased down to a few milliseconds, could effectively null the signal of the inversion recovery gradient-echo sequence with an echo time of 2.2 ms, obscuring the LGE [42].

At day 1, a significant decrease of *T*_2_^*^ (Fig. 4) and a transient depression of systolic performance (SWT; Fig. 6) were found not only in the infarct, but also to a lesser extent in the remote myocardium. This could be a secondary response in parallel to the structural changes in the infarct region. These findings are in agreement with those of Bogaert et al. [43] who observed a dysfunction in the remote area at 5 ± 2 days after reperfusion in patients with transmural anterior MI. Others have also shown that remodeling is initiated in the remote myocardium as early as 1 day after I/R injury [44–47].

Comparing Figs. 2 and 3 makes an interesting case. Figure 2 presented an example of a mouse with a considerable infarct at day 1, based on a large LGE positive area. However, at days 7 and 28, the infarct was difficult to detect on LGE, from which one could jump to the conclusion that a considerable part of the area at risk at day 1 recovered at later time points. However, the *T*_2_^*^ maps at day 7 and 28 revealed low *T*_2_^*^ values in the infarct area, indicating that the infarct was not recovered. The presence of non-viable myocardium (not detected by LGE) was confirmed by akinesia of that area and locally suppressed systolic thickening (SWT) in Cine imaging. Figure 3, conversely, showed one of three mice that presented a small infarct by LGE at day 1, but displayed no positive enhancement at day 7 and 28. For this mouse *T*_2_^*^ in the infarct region was preserved during follow-up, indicating that for this mouse the initial area at risk recovered at later time points, as confirmed by healthy LV function. Thus, *T*_2_^*^ is able to provide additional information, complementary to LGE, about the myocardial viability after infarction. Taken together, these findings suggest that *T*_2_^*^ can be used as a surrogate marker for myocardial recovery during follow-up after MI.

A limitation to the use of *T*_2_^*^ as a diagnostic marker for myocardial injury is contamination of intrinsic *T*_2_^*^ values by the presence of macroscopic susceptibility differences, mainly present at the air-lung interfaces [26, 27]. Occasionally, we observed lowered *T*_2_^*^ values at the lung-heart interface (see for example Fig. 3) in areas with no infarct and no wall-motion abnormalities. Estimations of infarct sizes on the basis of *T*_2_^*^ reduction were therefore inaccurate. Also the orientation of the heart with respect to the magnetic field could alter absolute *T*_2_^*^ values. Improvements in local shimming techniques might alleviate some of these susceptibility contaminations. Still, use of *T*_2_^*^ mapping as a stand-alone technique without the complementary information from LGE is undesired.

## Conclusions

Imaging of the myocardium with a *T*_2_^*^-mapping technique in a mouse model of myocardial I/R injury provides useful and complementary information to LGE and Cine imaging. The changes of the *T*_2_^*^ were associated with the infarct age, reversibility of the injury, and gave an indication of the structural variation in the myocardium throughout infarct maturation. *T*_2_^*^ decrease in the infarcts was associated with iron and collagen depositions. In cases where LGE was inconclusive, *T*_2_^*^ mapping was able to confirm the presence of a chronic myocardial scar in mice. Taken together, *T*_2_^*^ mapping may provide important additional information on myocardial viability in the acute and chronic phase and scar maturation after acute MI.
